# The relationship between psychological capital and self-directed learning ability among undergraduate nursing students—a cross-sectional study

**DOI:** 10.3389/fpsyg.2024.1413151

**Published:** 2024-09-04

**Authors:** Shanshan Ye, Wenyu Yue, Yixin Chen, Keying Gui, Yanlei Li, Runyi He, Xiaohong Liu

**Affiliations:** School of Medicine, Taizhou University, Taizhou, China

**Keywords:** nursing students, psychological capital, self-directed learning ability, optimism, resilience

## Abstract

**Background:**

Psychological capital has become a prominent focus in positive psychology, highlighting the positive influence of higher psychological capital on individuals. Self-directed learning ability is a fundamental skill for students, vital for enhancing academic performance and professional development, and is integral to the continuous learning process of nursing students. Recognizing the relationship between psychological capital and self-directed learning ability is crucial for the progress and development of undergraduate nursing students.

**Objective:**

This study aims to investigate the correlation between psychological capital and self-directed learning ability in undergraduate nursing students, as well as to explore the factors that influence these variables.

**Methods:**

A cross-sectional survey was conducted with 667 full-time undergraduate nursing students from a nursing school in Taizhou, China. Psychological capital and self-directed learning ability were assessed using the Psychological Capital Questionnaire and Self-Directed Learning Scale, respectively. Correlation and stepwise multiple regression analyses were then carried out to evaluate the relationship between psychological capital and self-directed learning ability among the participants.

**Results:**

The study revealed that the psychological capital score averaged at 103.24 ± 15.51, while the self-directed learning scale score averaged at 230.67 ± 27.66. Variations in psychological capital scores were noted based on factors including grade level, being an only child, growth environment, monthly living expenses, parental education level, voluntary selection of nursing major, and club experience. Similarly, differences in self-directed learning scores were associated with factors such as grade level, gender, parental education level, and voluntary selection of nursing major. Moreover, a positive correlation was identified between the overall psychological capital scores and the total self-directed learning ability scores among nursing students. Notably, the multiple regression analysis highlighted that optimism and resilience played significant roles as predictors of self-directed learning ability.

**Conclusion:**

Psychological capital is positively correlated with the self-directed learning ability of nursing students, with optimism and resilience identified as crucial predictors. Nursing educators can utilize strategies rooted in positive psychology and perseverance to improve the self-directed learning ability of nursing students.

## 1 Introduction

The nursing field increasingly emphasizes the necessity for nurses to engage in lifelong learning to develop adequate knowledge and practical skills, with the rapid advancement of medical knowledge and continuous updating of practice guidelines (Jin and Ji, 2021). Self-directed learning ability (SDL) is a core competence that enables students to enhance their academic achievement, clinical competence, and professional growth in nursing education (Park and Kim, 2023). It is considered a prerequisite for both nursing students and nurses to proactively address these demanding challenges (Cadorin et al., 2017). To better prepare nurses for professional practice, the nursing field is focusing on cultivating lifelong learning abilities (Bindon, 2017). Nursing students are expected to develop SDL habits and long-term learning skills during their education (Association, 2023).

Self-directed learning ability (SDL) is a process in which learners independently assess their learning needs, formulate learning goals, choose appropriate learning strategies, and evaluate learning outcomes without external assistance (Robinson and Persky, 2020). It includes aspects such as learning awareness, strategies, behavior, evaluation, and interpersonal relationships, all essential for lifelong learning (Premkumar et al., 2018). Iwasiw (1987) highlighted the cognitive similarities between SDL and nursing, albeit with different expressions. SDL has garnered increasing attention in higher education since the early 21st century (Levett-Jones, 2005). According to Knowles, a self-directed learner takes ownership of their learning, driven by internal motivation to develop, implement, and evaluate their approach to learning (Knowles, 1975). Concurrently, undergraduate nursing education is shifting towards learner-centered approaches (Valiga, 2012), emphasizing self-directed learning to enhance the quality of education (Robinson and Dearmon, 2013). Despite this trend, there is still ambiguity on how medical schools and postgraduate training programs should effectively implement and assess SDL educational interventions (Ricotta et al., 2022).

Psychological capital, a construct from positive psychology, is defined as a beneficial personality state that promotes growth and development (Luthans et al., 2007a). It encompasses four key dimensions: self-efficacy, hope, optimism, and resilience (Luthans and Youssef, 2004). This entails having the confidence (self-efficacy) to tackle challenges (Orgambidez et al., 2019), maintaining a positive outlook(optimistic) on present and future success (Bogler and Somech, 2019), persisting towards goals(hope), and sustaining or even reversing adversity (resilience) to achieve success (Karatepe and Avci, 2017; Manzanares et al., 2021). Individuals with high psychological capital are generally more motivated to pursue goals and better equipped to navigate challenges (Luthans et al., 2018; Newman et al., 2014).

The primary objective of this study is to investigate the relationship between psychological capital and SDL, providing new insights for improving self-directed learning ability. To enhance SDL, it is crucial to explore the factors that affect psychological capital and SDL. This research examines variables such as grade, gender, major, only-child status, growing environment, monthly living expenses, parental education, intention to choose a major, and club experience. Existing literature highlights the importance of these variables in relation to psychological capital and SDL. Studies reveal that only child tend to have better psychological well-being and higher levels of psychological capital compared to non-only children (Cao et al., 2021). Moreover, students from diverse growing environments exhibit varying levels of psychological capital (Terry et al., 2019). Parental education level (Hao et al., 2023), intention to choose a major (Jung, 2020), and club experience (Graner and Cerqueira, 2019) also influence nursing students’ psychological capital to varying degrees.

Mainland Chinese medical students have been found to exhibit a moderate level of SDL (Yang et al., 2021). Factors such as age, gender, family income, learning resources, and place of residence have shown significant correlations with the overall SDL score (Malekian et al., 2015; Yang et al., 2021). The professional identity of health students has been positively linked to their SDL (Xu et al., 2024). In the case of Vietnamese undergraduate nursing students, SDL is influenced by factors like residence, academic burden, and availability of learning materials (Thu et al., 2024). Gender also plays a role, with male nursing students tending to display higher levels of SDL (Lee et al., 2020). Furthermore, stressors related to academics, employment, further education, and life circumstances can deplete psychological resources, potentially leading to learning burnout and a decline in SDL (Yang et al., 2024).

This study aims to explore the relationship between psychological capital and self-directed learning (SDL) among undergraduate nursing students. By analyzing these variables, we aim to gain a deeper understanding of the factors influencing psychological capital and SDL in this population. The findings of this research will contribute to enhancing the SDL of nursing students and ultimately improve the quality of nursing education, better preparing future nursing professionals for their practice.

### 1.1 The effects of psychological capital on self-directed learning ability

Psychological capital is a construct that can be cultivated and enhanced over time (Jiang et al., 2023). Effective development interventions for psychological capital require appropriate contextual frameworks (Luthans and Youssef-Morgan, 2017). Individuals with higher psychological capital are better equipped to cope with stress and face challenges (Elliott and Fry, 2021; Karatepe and Avci, 2017). As a positive psychological characteristic, psychological capital strengthens an individual’s abilities and increases the resources to overcome difficult situations and achieve success (Guo et al., 2021).

Scientific management of positive psychological capital significantly contributes to mental health (Dwyer et al., 2019), stress coping behaviors (Mubarak et al., 2021), and academic performance (Luthans et al., 2012). Incorporating psychological capital development into curriculum design, pedagogical approaches, and coursework can enhance academic performance by fostering psychological capital growth (Luthans et al., 2012).

We argue that students with high levels of psychological capital are more likely to exhibit strong self-directed learning ability. In light of these arguments, we propose the following:

*Hypothesis 1*: Psychological capital is positively related to self-directed learning abilities.

### 1.2 Predictive effects of various dimensions of psychological capital on self-directed learning ability

Psychological capital, comprising self-efficacy, hope, optimism and resilience, plays a crucial role in fostering self-directed learning ability. Self-efficacy influences an individual’s motivation and learning goals (Wu et al., 2020), with high self-efficacy leading to increased willingness to tackle challenging tasks and explore optimal learning methods (Kong et al., 2021). Resilient individuals, adept at managing stress and setbacks, exhibit enhanced self-directed learning abilities by persistently pursuing their learning objectives (Luthans et al., 2015). Additionally, resilience contributes to cognitive flexibility and problem-solving skills, essential components of SDL (Moenkemeyer et al., 2012). Research also suggests a positive correlation between optimism, emotional intelligence, and self-directed learning ability (Hwang and Kim, 2023). Based on these insights, we propose the following:

*Hypothesis 2*: The dimensions of psychological capital (self-efficacy, hope, optimism, and resilience) serve as significant predictors of self-directed learning ability.

## 2 Objects and methods

### 2.1 Participants and procedure

This study employed a whole-group sampling method. Questionnaires were distributed to 704 full-time undergraduate nursing students from freshman to senior year in the Department of Nursing (including nursing and midwifery) at a medical school in Taizhou, China, between October and November 2022. A total of 667 valid questionnaires were returned, resulting in an effective rate of 94.74%. Samples with missing or incomplete answers were excluded from the analysis. Prior to questionnaire completion, the researcher provided an explanation of the study’s purpose and content to the participants. Informed consent was obtained from all participants, and any personal information collected was solely for research purposes.

### 2.2 Measures

#### 2.2.1 General information questionnaire

A general information questionnaire designed by the researchers includes questions regarding grade, gender, age, major, only child, growth environment, monthly living expenses, parental education levels, intention to choose a major, part-time experience, and club experience.

#### 2.2.2 Psychological capital questionnaire

The Psychological Capital Questionnaire, developed by Luthans et al. (2007b), has a Chinese version (PCQ-24) developed by Li (Luthans et al., 2008). It consists of 24 items categorized into four dimensions (Luthans and Youssef, 2004): Self-efficacy (items 1–6), hope (items 7–12), resilience (items 13–18), and optimism (items 19–24). Each item in the scale is rated on a 6-point Likert scale, ranging from “Strongly Disagree” to “Strongly Agree,” with scores from 1 to 6 assigned accordingly. Items 13, 20, and 23 involve reverse scoring, and the total score ranges from 24 to 144. A higher score indicates greater psychological capital (Zhong et al., 2013). The Cronbach’s alpha for this questionnaire is 0.912, with each dimension ranging from 0.738 to 0.924, indicating good reliability. The validation factor analysis indicators include X2/df = 1.805, NFI = 0.929, RFI = 0.884, IFI = 0.967, TLI = 0.945, CFI = 0.966, and RMSEA = 0.062, demonstrating satisfactory validity (Luo and He, 2010).

#### 2.2.3 Self-directed learning scale

Self-Directed Learning Scale (SDLS) was developed by Professor Williamson in 2007 and subsequently tested for reliability and validity in a study involving nursing students at Thames Valley University (Cadorin et al.). The scale comprises 60 items across 5 dimensions: learning awareness (items 1–12), learning strategies (items 13–24), learning behavior (items 25–36), learning evaluation (items 37–48), interpersonal relationships (items 49–60). Scoring is done using a Likert 5-point scale, with a total score range of 60–300. Scores between 60–140 are categorized as ‘Low’,141–220 as ‘Medium’, and 221–300 as ‘High’, with higher scores indicating better self-directed learning ability. This scale distinguishes itself by fully capturing the essence of self-directed learning and offering insights into respondents’ SDL characteristics through dimensional analysis, enabling targeted interventions for cultivating SDL. The Chinese version of SDLS, developed by Shen (2011), demonstrated strong reliability and validity in statistical testing, with a Cronbach alpha of 0.966, retest reliability of 0.855, and CVI of 0.963, affirming its adequacy in measurement.

### 2.3 Data collection

Questionnaires were distributed online using Questionnaire Star (an APP). Prior to distribution, researchers provided a unified explanation of the questionnaire’s purpose in a clear and concise language. The survey aimed to assess students’ psychological capital and self-directed learning ability to inform the development of teaching strategies. Participants were instructed to respond truthfully based on their individual circumstances. Data collected will be used solely for scientific analysis and will not disclose any personal information. Students completed the form within a 20-min timeframe. Informed consent was obtained from all participants.

### 2.4 Data analysis

SPSS 22.0 was utilized for data input and statistical analysis. Descriptive statistical findings were presented using measures such as mean, standard deviation, rate, and composition ratio. *T*-tests and analysis of variance were used to explore the factors influencing self-directed learning ability and psychological capital. The correlation between scores for psychological capital and self-directed learning ability was examined through Pearson correlation and multiple stepwise regression analysis. A significance level of *p* < 0.05 was considered statistically significant.

## 3 Results

### 3.1 General information about the participants

The average age of the participants was (19.70 ± 1.70) years. The descriptive statistical results of the sample were presented in Table 1.

**Table 1:** General information about the participants.

**Item**		**n**	**%**
Grades	1	237	35.53
	2	161	24.14
	3	154	23.09
	4	115	17.24
Gender	Male	90	13.49
	Female	577	86.51
Major	Nursing	476	71.36
	Midwifery	191	28.64
Only child	Yes	200	29.99
	No	467	70.01
Growth environment	City	135	20.24
	Villages and towns	137	20.54
	Country	395	59.22
Monthly living expenses	500–1,000	49	7.35
	1,000–1,500	256	38.38
	1,500–2000	263	39.43
	>2000	99	14.84
Father’s education	Junior high school or below	422	63.27
	Senior high school or technical secondary school	184	27.59
	Professional training college or above	61	9.15
Mother’s education	Junior high school or below	464	69.57
	Senior high school or technical secondary school	153	22.94
	Professional training college or above	50	7.50
Intention to choose a major	Yes	505	75.71
	No	162	24.29
Part-time experience	Yes	211	31.63
	No	456	68.37
Club experience	Yes	601	90.10
	No	66	9.90

### 3.2 Current status of psychological capital and self-directed learning ability of undergraduate nursing students

The study involved 667 nursing students, with an average psychological capital score of 103.24 ± 15.51. The individual scores for self-efficacy, hope, resilience, and optimism were 26.43 ± 4.33, 25.64 ± 4.57, 25.65 ± 4.19, and 25.53 ± 4.30, respectively. Additionally, the students had a self-directed learning ability score of 230.67 ± 27.66. The scores for learning awareness, learning strategies, learning behavior, learning evaluation, and interpersonal relationships were 46.32 ± 6.00, 46.11 ± 5.97, 44.50 ± 6.70, 46.98 ± 5.95, and 46.76 ± 6.00, respectively.

### 3.3 Comparison of psychological capital and self-directed learning ability among nursing undergraduates with different characteristics

The results of the one-way analysis of variance revealed statistically significant differences in psychological capital scores across grades (*F* = 4.42, *p* < 0.01), only child (*t* = 3.36, *p* < 0.01), growth environment (*F* = 3.10, *p* < 0.05), monthly living expenses (*F* = 5.63, *p* < 0.01), father’s education (*F* = 6.58, *p* < 0.01), mother’s education (*F* = 7.37, *p* < 0.01), intention to choose a major (*t* = 4.71, *p* < 0.01) and club experience (*t* = 2.18, *p* < 0.05).

Similarly, differences in self-directed learning scores across grades (*F* = 4.86, *p* < 0.01), gender (*t* = 3.68, *p* < 0.01), father’s education (*F* = 3.99, *p* < 0.05), mother’s education (*F* = 3.20, *p* < 0.05) and intention to choose a major (*t* = 2.30, *p* < 0.05) were also found to be statistically significant. See Table 2. Detailed multiple comparisons of psychological capital and self-directed learning ability can be found in Tables 3–10.

**Table 2:** Comparison of psychological capital and self-directed learning ability among nursing students with different characteristics.

**Item**	***n* (%)**	**Psychological capital score**	**t/F**	***p***	**Self-directed learning ability score**	**t/F**	***p***
Grades							
1	237(35.5)	105.27 ± 17.19	4.42	0.00^**^	235.11 ± 32.18	4.86	0.00^**^
2	161(24.1)	104.61 ± 14.52			231.88 ± 23.70		
3	154(23.1)	100.95 ± 14.06			225.11 ± 24.79		
4	115(13.5)	100.21 ± 14.33			227.23 ± 24.86		
Gender							
Male	90(13.5)	106.06 ± 17.76	1.85	0.06	240.01 ± 31.63	3.48	0.00^**^
Female	577(86.5)	102.80 ± 15.10			229.20 ± 26.73		
Major							
Nursing	476(71.4)	103.41 ± 14.93	0.43	0.67	229.67 ± 26.82	−1.46	0.15
Midwifery	191(28.6)	102.83 ± 16.92			233.13 ± 29.59		
Only child							
Yes	200(30.0)	106.30 ± 15.41	3.36	0.00^**^	233.53 ± 27.11	1.75	0.08
No	467(70.0)	101.93 ± 15.38			229.43 ± 27.84		
Growth environment							
City	135(20.2)	105.66 ± 16.62	3.10	0.04^*^	233.81 ± 26.82	2.19	0.11
Villages and towns	137(20.5)	101.00 ± 14.34			226.86 ± 26.50		
Country	395(59.3)	103.19 ± 15.43			230.91 ± 28.25		
Monthly living expenses							
500–1,000	49(7.3)	100.73 ± 16.84	5.63	0.00^**^	231.65 ± 28.18	1.69	0.17
1,000–1,500	256(38.4)	101.19 ± 14.12			229.31 ± 25.33		
1,500–2000	263(39.4)	103.81 ± 15.64			229.68 ± 26.95		
>2000	99(14.9)	108.28 ± 16.83			236.26 ± 34.08		
Father’s education							
Junior high school or below	422(63.3)	102.71 ± 14.68	6.58	0.00^**^	229.92 ± 26.18	3.68	0.03^*^
Senior high school or technical secondary school	184(27.6)	102.21 ± 16.10			229.36 ± 26.80		
professional training college or above	61(9.1)	110.02 ± 17.77			239.75 ± 37.47		
Mother’s education							
Junior high school or below	464(69.6)	102.03 ± 14.97	7.37	0.00^**^	229.17 ± 26.19	3.20	0.04^*^
Senior high school or technical secondary school	153(22.9)	104.58 ± 16.60			232.54 ± 31.05		
Professional training college or above	50(7.5)	110.36 ± 15.07			238.78 ± 28.81		
Intention to choose a major							
Yes	505(75.7)	104.82 ± 15.03	4.71	0.00^**^	232.06 ± 27.46	2.30	0.02^*^
No	162(24.3)	98.33 ± 16.01			226.31 ± 27.92		
Part-time experience							
Yes	211(31.6)	102.44 ± 14.40	−0.91	0.36	231.43 ± 24.08	0.52	0.60
No	456(68.4)	103.61 ± 16.00			230.31 ± 29.19		
Club experience							
Yes	601(90.1)	103.67 ± 15.06	2.18	0.03^*^	231.06 ± 26.24	0.84	0.40
No	66(9.9)	99.3 ± 18.84			227.00 ± 38.34		

*p < 0.05; **p < 0.01.

**Table 3:** Multiple comparisons of psychological capital scores of nursing students in different grades.

(I) Grades	(J) Grades	Mean difference (I-J)	Std. error	*p*	95% Confidence interval	
					Lower bound	Upper bound
4	3	−0.746	1.897	0.694	−4.47	2.98
	2	−4.400^*^	1.879	0.02^*^	−8.09	−0.71
	1	−5.061^**^	1.749	0.004^**^	−8.5	−1.63
3	4	0.746	1.897	0.694	−2.98	4.47
	2	−3.654^*^	1.735	0.036^*^	−7.06	−0.25
	1	−4.315^*^	1.593	0.007^**^	−7.44	−1.19
2	4	4.400^*^	1.879	0.02^*^	0.71	8.09
	3	3.654^*^	1.735	0.036^*^	0.25	7.06
	1	−0.661	1.572	0.674	−3.75	2.43
1	4	5.061^**^	1.749	0.004^**^	1.63	8.5
	3	4.315^**^	1.593	0.007^**^	1.19	7.44
	2	0.661	1.572	0.674	−2.43	3.75

*p < 0.05; **p < 0.01.

**Table 4:** Multiple comparisons of psychological capital scores of nursing students from different growth environments.

(I) Growth environments	(J) Growth environments	Mean difference (I-J)	Std. error	*p*	95% Confidence interval	
					Lower bound	Upper bound
City	Villages and towns	4.667^**^	1.875	0.01^**^	0.98	8.35
	Country	2.464	1.542	0.11	−0.56	5.49
Villages and towns	City	−4.667^**^	1.875	0.01^**^	−8.35	−0.98
	Country	−2.202	1.533	0.15	−5.21	0.81
Country	City	−2.464	1.542	0.11	−5.49	0.56
	Villages and towns	2.202	1.533	0.15	−0.81	5.21

*p < 0.05; **p < 0.01.

**Table 5:** Multiple comparisons of psychological capital scores of nursing students with different monthly living expenses.

(I) Monthly living expenses	(J) Monthly living expenses	Mean Difference (I-J)	Std. error	*p*	95% Confidence interval	
					Lower bound	Upper bound
500–1,000	1,000–1,500	−0.457	2.394	0.849	−5.16	4.24
	1,500–2000	−3.071	2.389	0.199	−7.76	1.62
	>2000	−7.548^**^	2.682	0.005^**^	−12.81	−2.28
1,000–1,500	500–1,000	0.457	2.394	0.849	−4.24	5.16
	1,500–2000	−2.615	1.348	0.053	−5.26	0.03
	>2000	−7.091^**^	1.817	0.000^**^	−10.66	−3.52
1,500–2000	500–1,000	3.071	2.389	0.199	−1.62	7.76
	1,000–1,500	2.615	1.348	0.053	−0.03	5.26
	>2000	−4.477^*^	1.81	0.014^*^	−8.03	−0.92
>2000	500–1,000	7.548^**^	2.682	0.005^**^	2.28	12.81
	1,000–1,500	7.091^*^	1.817	0.000^**^	3.52	10.66
	1,500–2000	4.477^*^	1.81	0.014^*^	0.92	8.03

*p < 0.05; **p < 0.01.

**Table 6:** Multiple comparisons of psychological capital scores of nursing students with different levels of father education.

(I). Father’s education	(J). Father’s education	Mean difference (I-J)	Std. error	*P*	95% Confidence interval	
					Lower bound	Upper bound
Junior high school or below	Senior high school or technical secondary school	0.499	1.359	0.714	−2.17	3.17
	Professional training college or above	−7.305^**^	2.107	0.001^**^	−11.44	−3.17
Senior high school or technical secondary school	Junior high school or below	−0.499	1.359	0.714	−3.17	2.17
	Professional training college or above	−7.804^**^	2.273	0.001^**^	−12.27	−3.34
Professional training college or above	Junior high school or below	7.305^**^	2.107	0.001^**^	3.17	11.44
	Senior high school or technical secondary school	7.804^**^	2.273	0.001^**^	3.34	12.27

*p < 0.05; **p < 0.01.

**Table 7:** Multiple comparisons of psychological capital scores of nursing students with different levels of mother education.

(I) Mother’s education	(J) Mother’s education	Mean difference (I-J)	Std. error	*P*	95% Confidence interval	
					Lower bound	Upper bound
Junior high school or below	Senior high school or technical secondary school	−2.541	1.432	0.077	−5.35	0.27
	Professional training college or above	−8.326^**^	2.287	0.000^**^	−12.82	−3.83
Senior high school or technical secondary school	Junior high school or below	2.541	1.432	0.077	−0.27	5.35
	Professional training college or above	−5.785^*^	2.503	0.021^*^	−10.7	−0.87
Professional training college or above	Junior high school or below	8.326^**^	2.287	0.000^**^	3.83	12.82
	Senior high school or technical secondary school	5.785^*^	2.503	0.021^*^	0.87	10.7

*p < 0.05; **p < 0.01.

**Table 8:** Multiple comparisons of self-directed learning ability score of nursing students in different grades.

(I) Grades	(J) Grades	Mean Difference (I-J)	Std. error	*P*	95% Confidence interval	
					Lower bound	Upper bound
4	3	4.258	6.104	0.486	−7.73	16.24
	2	−8.202	6.047	0.175	−20.08	3.67
	1	−13.807^*^	5.629	0.014^*^	−24.86	−2.75
3	4	−4.258	6.104	0.486	−16.24	7.73
	2	−12.461^*^	5.583	0.026^*^	−23.42	−1.5
	1	−18.066^**^	5.127	0.000^**^	−28.13	−8
2	4	8.202	6.047	0.175	−3.67	20.08
	3	12.461^*^	5.583	0.026^*^	1.5	23.42
	1	−5.605	5.059	0.268	−15.54	4.33
1	4	13.807^*^	5.629	0.014^*^	2.75	24.86
	3	18.066^**^	5.127	0.000^**^	8	28.13
	2	5.605	5.059	0.268	−4.33	15.54

*p < 0.05; **p < 0.01.

**Table 9:** Multiple comparisons of self-directed learning ability score of nursing students with different levels of father education.

(I) Father’s education	(J) Father’s education	Mean difference (I-J)	Std. error	*P*	95% Confidence interval	
					Lower bound	Upper bound
Junior high school or below	Senior high school or technical secondary school	1.088	4.394	0.804	−7.54	9.72
	professional training college or above	−18.464^**^	6.813	0.007^**^	−31.84	−5.09
Senior high school or technical secondary school	Junior high school or below	−1.088	4.394	0.804	−9.72	7.54
	professional training college or above	−19.552^**^	7.348	0.008^**^	−33.98	−5.12
Professional training college or above	Junior high school or below	18.464^**^	6.813	0.007^**^	5.09	31.84
	Senior high school or technical secondary school	19.552^**^	7.348	0.008^**^	5.12	33.98

*p < 0.05; **p < 0.01.

**Table 10:** Multiple comparisons of self-directed learning ability score of nursing students with different levels of mother education.

(I) Mother’s education	(J) Mother’s education	Mean Difference (I-J)	Std. error	*P*	95% Confidence interval	
					Lower bound	Upper bound
Junior high school or below	Senior high school or technical secondary school	−5.619	4.643	0.227	−14.74	3.5
	Professional training college or above	−17.237^*^	7.413	0.02^*^	−31.79	−2.68
Senior high school or technical secondary school	Junior high school or below	5.619	4.643	0.227	−3.5	14.74
	Professional training college or above	−11.619	8.113	0.153	−27.55	4.31
Professional training college or above	Junior high school or below	17.237^*^	7.413	0.02^*^	2.68	31.79
	Senior high school or technical secondary school	11.619	8.113	0.153	−4.31	27.55

*p < 0.05; **p < 0.01.

The results in Table 3 demonstrate notable variations in psychological capital scores among nursing students across various grades. Specifically, students in grade 1 and grade 2 exhibit higher psychological capital scores in comparison to students in grades 3 and 4.

The results presented in Table 4 demonstrate notable variations in psychological capital scores among nursing students based on their respective growth environments. Specifically, students hailing from cities exhibit significantly higher psychological capital scores in comparison to their counterparts from villages and towns. Interestingly, no significant disparities were observed between students from rural countryside areas and those from cities or villages and towns.

The findings suggest a notable disparity in psychological capital scores among nursing students based on their monthly living expenses. Specifically, students with higher monthly living expenses demonstrate significantly higher psychological capital scores when compared to their counterparts with lower living expenses.

The results in Tables 6, 7 demonstrate notable variations in psychological capital scores among nursing students based on the educational levels of their parents. It is evident that students with parents who have attained higher education levels exhibit notably higher psychological capital scores in comparison to students with parents who have lower education levels.

The results in Table 8 illustrate significant differences in self-directed learning ability scores among nursing students across various grade levels. Notably, students in grades 3 and 4 demonstrate markedly higher self-directed learning ability scores compared to their counterparts in grades 1 and 2.

The results in Tables 9, 10 illustrate significant variations in self-directed learning ability scores among nursing students, based on the educational levels of their parents. It is evident that students with parents holding higher education levels generally exhibit notably higher self-directed learning ability scores.

### 3.4 Correlation between psychological capital score and self-directed learning ability score

The results presented in Table 11 indicate a positive correlation between the total psychological capital score and its dimensions with the self-directed learning ability score. A stepwise multiple regression analysis was conducted with general information, psychological capital score, and its four dimensions as independent variables, and self-directed learning ability score as the dependent variable. The analysis revealed that psychological capital score, gender, major, optimism, and resilience significantly influenced the self-directed learning ability scores, collectively explaining 51.8% of the total variation. Refer to Table 12 for more details.

**Table 11:** Correlation analysis of psychological capital scores, self-directed learning scores, and each dimension.

Item	1	2	3	4	5	6	7	8	9	10	11
(1) PCQ score	1										
(2) Self-efficacy	0.87^**^	1									
(3) Hope	0.92^**^	0.76^**^	1								
(4) Resilience	0.91^**^	0.71^**^	0.80^**^	1							
(5) Optimism	0.87^**^	0.63^**^	0.72^**^	0.74^**^	1						
(6) SDL score	0.71^**^	0.63^**^	0.67^**^	0.63^**^	0.59^**^	1					
(7) Awareness	0.70^**^	0.61^**^	0.68^**^	0.62^**^	0.60^**^	0.89^**^	1				
(8) Strategies	0.61^**^	0.56^**^	0.58^**^	0.53^**^	0.51^**^	0.90^**^	0.78^**^	1			
(9) Behavior	0.62^**^	0.54^**^	0.60^**^	0.56^**^	0.50^**^	0.91^**^	0.75^**^	0.78^**^	1		
(10) Evaluation	0.62^**^	0.57^**^	0.58^**^	0.55^**^	0.51^**^	0.91^**^	0.76^**^	0.76^**^	0.78^**^	1	
(11) Relationships	0.65^**^	0.59^**^	0.61^**^	0.60^**^	0.54^**^	0.91^**^	0.75^**^	0.75^**^	0.77^**^	0.83^**^	1

*p < 0.05; **p < 0.01.

**Table 12:** Self-directed learning ability score stepwise multiple regression analysis.

Model		Unstandardized coefficients		Standardized coefficients	*t*	*p*
		B	Std. error	Beta		
1	(Constant)	100.32	5.10		19.67	0.00
	Psychological capital score	1.26	0.05	0.71	25.85	0.00
2	(Constant)	113.97	6.76		16.85	0.00
	Psychological capital score	1.25	0.05	0.70	25.72	0.00
	Gender	−6.73	2.21	−0.08	−3.05	0.00
3	(Constant)	109.66	6.85		16.01	0.00
	Psychological capital score	1.25	0.05	0.70	25.91	0.00
	Gender	−8.18	2.24	−0.10	−3.65	0.00
	Major	5.42	1.69	0.09	3.21	0.00
4	(Constant)	111.04	6.86		16.19	0.00
	Psychological capital score	1.43	0.10	0.80	14.96	0.00
	Gender	−8.63	2.24	−0.11	−3.85	0.00
	Major	5.50	1.68	0.09	3.27	0.00
	Optimism	−0.76	0.35	−0.12	−2.19	0.03
5	(Constant)	111.49	6.85		16.29	0.00
	Psychological capital score	1.69	0.16	0.95	10.63	0.00
	Gender	−8.89	2.24	−0.11	−3.97	0.00
	Major	5.83	1.69	−0.10	3.46	0.00
	Optimism	−0.92	0.36	−0.14	−2.60	0.01
	Resilience	−0.90	−0.44	−0.14	−2.04	0.04

*R*^2^ = 0.522, adjust *R*^2^ = 0.518, *F* = 144.42, *p* < 0.001.

## 4 Discussion

The primary aim of this study was to assess the levels of psychological capital and self-directed learning abilities in nursing undergraduates, analyze influencing factors and their relationships, and suggest strategies for improving SDL. These objectives are crucial for enhancing the quality of nursing education and preparing nursing professionals for their future roles.

### 4.1 Factors influencing psychological capital of nursing students

#### 4.1.1 Grade

Lower-grade nursing students exhibit high levels of psychological capital, while higher-grade students face the challenge of mastering complex medical courses alongside concerns about academic performance and heavy workloads. This pressure can lead to burnout and impact the mental health (Zhou et al., 2022). As students progress to clinical practice, they face increased stress from balancing theoretical knowledge with practical experience. Research indicates that nursing students at this stage often experience anxiety related to decision-making, employment prospects, graduation projects, licensure exams, and clinical competence (Yi et al., 2022). This anxiety can lead to decreased resilience and happiness, ultimately affecting their psychological capital. Interventions specifically tailored to enhance psychological capital are crucial for higher-grade nursing students to navigate the challenges they face and build resilience for their post-graduation transition (Liu X et al., 2022).

#### 4.1.2 Only child

Only child exhibits a healthier psychological state and higher psychological capital compared to non-only child, aligning with prior research findings (Cao et al., 2021). Being the focal point of a family, only child tends to have more effective interactions and communication with parents, leading to stronger parent–child relationships (Cao et al., 2021). This closer bond with parents can foster a greater sense of confidence and security. Conversely, parents in non-only child families may struggle to establish close relationships with each child and lack social support (Sun et al., 2022). The increase in the number of children may lead to a diversion of resources within the family. The unique aspect of being an only child lies in the fact that they receive undivided attention from their parents, similar to a firstborn, and they also experience the security of never being displaced by a younger sibling, akin to a lastborn (Chu et al., 2015). Therefore, the only child receives concentrated family resources and parents invest more energy into their growth. When faced with difficulties, the only child tends to perceived lower levels of pressure and may adopt a more positive coping style (Cao et al., 2021), leading to a more stable psychological state and stronger resilience. Consequently, the only child tends to possess a more robust psychological capital.

#### 4.1.3 Growing environment

Previous research has demonstrated that urban students exhibit notably higher levels of general self-efficacy and nursing self-efficacy compared to their rural counterparts (Terry et al., 2019). Urban students typically have access to diverse opportunities for self-efficacy development, whereas rural students often face limited social support and heightened life stress, resulting in lower levels of optimism and hope (Liu et al., 2021; Tong et al., 2019). However, the findings of this study solely suggest a distinction in psychological capital between urban and township students, with no significant difference observed between rural and urban students. This discrepancy may be attributed to the relatively affluent economic status of rural regions in the area.

#### 4.1.4 Monthly living expenses

Psychological capital is influenced by the monthly living expenses of students. Research suggests that students with higher living expenses tend to exhibit greater levels of optimism and hope (Gan and Wang, 2021). A study conducted in Australia found that inadequate monthly living expenses due to financial constraints are a significant source of stress for nursing students (He et al., 2018). Higher living costs and lower disposable income often force students to take on part-time jobs to sustain their living standards, cover study expenses, and even pay mortgages. When students struggle to balance intense study commitments with work, their stress levels increase (He et al., 2018), leading to reduced satisfaction and optimism in life, ultimately resulting in lower psychological capital (Arias-de la Torre et al., 2019).

#### 4.1.5 Parental education level

Nursing students with higher parental education demonstrated increased psychological capital. Parents with higher levels of education possess greater knowledge about mental health (Hao et al., 2023), enabling them to effectively guide their children in managing emotions and regulating psychology. Furthermore, when children confide in their parents during times of stress, those with higher education can offer more effective support (Rafati et al., 2020). Consequently, offspring of highly educated parents are more inclined to prioritize mental health, exhibit elevated psychological capital, and better navigate stress and frustration in nursing studies (Hamaideh et al., 2017).

#### 4.1.6 Intention to choose a major

Nursing students who voluntarily choose nursing as their major exhibit higher psychological capital compared to those who involuntarily choose nursing. This difference may be attributed to the stronger sense of professional identity and higher professional satisfaction among students who make a voluntary choice (Jung, 2020). Previous studies (Liu J et al., 2022) have indicated that students with a stronger professional identity tend to have higher self-confidence and a more optimistic outlook, leading to increased motivation for learning and clinical practice. Additionally, students who make voluntary choices experience less role stress (Sun et al., 2016), are less prone to negative emotions and burnout, and require less social support (Liu J et al., 2022). As a result, students who voluntarily choose the nursing program tend to have higher psychological capital and demonstrate a more positive, confident, and optimistic attitude towards professional challenges.

#### 4.1.7 Club experience

Nursing students with club experience demonstrate higher levels of resilience, which plays a protective role in maintaining the mental health of nursing students when dealing with stress (Graner and Cerqueira, 2019). Conversely, nursing students without club experience tend to experience increased isolation and distress when faced with stress, and exhibit lower levels of psychological capital, possibly due to a lack of social support from clubs (Silva et al., 2014).

### 4.2 Factors influencing self-directed learning ability of nursing students

#### 4.2.1 Grade

The study found that first-year nursing students exhibit higher levels of internal and external motivation (Kim and Yang, 2020) compared to third and fourth-year students, making them more prepared and motivated for self-directed learning. In contrast, intrinsic motivation tends to decrease as students progress through higher grade levels, impacting their learning abilities and resulting in lower self-directed learning levels (Grande et al., 2022). Additionally, newly enrolled students demonstrate higher levels of positive emotions, interest in learning, willpower, strategic thinking skills, self-regulation, and self-learning skills (Schweder and Raufelder, 2019). First-year students also benefit from more flexible schedules, allowing them to delve deeper into their studies and pursue their interests (Yang et al., 2021). The reduced self-directed learning ability among senior students could be attributed to the pressures of internships and the limited time available for self-directed learning.

#### 4.2.2 Gender

Research studies have indicated that males generally exhibit higher self-directed learning abilities compared to females. Surveys (Kar et al., 2014) have demonstrated that males display a greater inclination towards independent learning, leveraging their strengths in self-management and self-control. They exhibit higher levels of enthusiasm and confidence in nursing, and are more inclined towards independent learning (Lee et al., 2020). Men can better utilize their learning ability to acquire and process information, as well as their strengths in rational thinking in school (Wong et al., 2021).

#### 4.2.3 Intention to choose a major

Research (Yang et al., 2021) indicates that students who are highly motivated in their chosen majors tend to be more adept at independent learning. Additionally, students who are motivated in selecting their majors demonstrate improved problem-solving skills, as well as heightened interest and enthusiasm for learning. A strong inclination towards their majors enables students to exhibit greater self-confidence, establish effective learning goals and habits, and achieve higher GPAs. Such students develop a deep passion for their majors, which in turn boosts their motivation for learning and encourages proactive engagement in independent study. Consequently, students with strong aspirations for their majors exhibit enhanced self-directed learning abilities.

### 4.3 The relationship between psychological capital and self-directed learning ability

Optimism is a positive emotion that is strongly linked to self-directed learning ability (Chang et al., 2021). Optimism plays a vital role in learning (Li et al., 2021). From an optimist’s perspective, adverse events are seen as transient, caused by external factors, and specific to a particular situation, while positive events are viewed as connected to internal factors that are more permanent and recurrent. Optimists are more likely to possess higher self-directed learning abilities (Hwang and Kim, 2023). This is because optimistic individuals tend to approach challenges with a problem-solving mindset, maintain motivation, and persist in the face of difficulties, all of which are crucial for effective self-directed learning ability (Lopes and Cunha, 2008).

The positive role of resilience in work and learning has been extensively documented (Caniëls et al., 2022). Resilience is a dynamic process that influences individuals’ ability to adapt to adversity at any level of functioning (VanMeter and Cicchetti, 2020). Higher resilience is associated with improved better memory performance (Lin et al., 2017), crucial for learning and information retention. Individuals with high resilience tend to experience lower psychological stress (Bacchi and Licinio, 2017), enabling them to sustain focus and motivation in their academic pursuits. Consequently, those with high resilience are more likely to possess enhanced self-directed learning abilities as they can effectively manage stress, recover from setbacks, and continue to pursue their learning goals with determination (Luthans et al., 2015).

Resilience has been found to improve cognitive flexibility and problem-solving skills, both essential for self-directed learning ability (Moenkemeyer et al., 2012). Those who are resilient excel at setting achievable goals, tracking their progress, and adjusting their strategies as needed, all of which contribute to their ability to learn independently (Trigueros et al., 2019).

The impact of optimism and resilience on self-directed learning ability is evident through their ability to enhance motivation, reduce stress, and improve cognitive functions. These findings emphasize the significance of nurturing psychological capital in educational environments to enhance self-directed learning ability effectively.

## 5 Conclusion

The determinants of psychological capital include grade, being an only child, growing environment, monthly living expenses, parental education level, intention to choose a major and club experience. Similarly, factors influencing self-directed learning ability include grade, gender, and the intention to choose a major. There is a significant positive correlation between psychological capital and self-directed learning ability, with optimism and resilience being key factors influencing the latter. It is recommended that nursing educators incorporate positive psychology teaching methods to boost students’ levels of optimism, while hospitals offering clinical placements for nursing students should prioritize psychological interventions to enhance resilience.

### 5.1 Limitation

For this study, it is important to acknowledge certain limitations. Firstly, the sample used in our research is limited geographically and therefore may not provide a comprehensive overview of the phenomenon across the entire country. Secondly, there is a potential for bias in the results due to the significant gender disparity in the nursing major and the small number of male nursing students included in the study. Thirdly, as we employed a cross-sectional study approach, the results reflect only a snapshot in time. Lastly, some of the students in our sample were impacted by the COVID-19 pandemic, which could have influenced their learning approach and overall academic performance, a factor that has not been adequately addressed.

To further explore the relationship between psychological capital and self-directed learning ability, a larger and more diverse sample is necessary. Future studies should consider utilizing a longitudinal research design and randomized sampling methods. Additionally, the effects of the covid-19 pandemic on students’ learning experiences should be carefully examined.

### 5.2 Practical implications

This study offers valuable insights for nurse educators in developing strategies to enhance nursing students’ psychological capital and self-directed learning ability. Nursing students with higher psychological capital demonstrate improved career adaptability (Sun et al., 2023) and motivation for self-directed learning. Additionally, resilience plays a key role in maintaining or quickly restoring psychological well-being (Kunzler et al., 2020), ultimately boosting self-efficacy, hope (Atitsogbe et al., 2019; Pajic et al., 2018; Rivera et al., 2021). Therefore, it is crucial to implement comprehensive intervention strategies aimed at enhancing nursing students’ self-directed learning ability by elevating their psychological capital.

We suggest that nursing educators utilize positive psychology approaches to bolster the psychological capital of nursing students and employ psychological intervention methods to enhance resilience. For nursing students transitioning from school to clinical practice, we recommend that managers enhance psychometric evaluations to accurately assess levels of psychological capital (Dawkins et al., 2013). This improvement is essential for the successful implementation of targeted interventions. Furthermore, organizing interns to participate in psychological courses regularly to implement interventions that enhance clinical resilience is also encouraged.

## Data Availability

The original contributions presented in the study are included in the article/supplementary material, further inquiries can be directed to the corresponding author/s.
